# Long contiguous stretches of homozygosity detected by chromosomal microarrays (CMA) in patients with neurodevelopmental disorders in the South of Brazil

**DOI:** 10.1186/s12920-019-0496-5

**Published:** 2019-03-12

**Authors:** Tiago Fernando Chaves, Luan Freitas Oliveira, Maristela Ocampos, Ingrid Tremel Barbato, Gisele Rozone de Luca, Jorge Humbeto Barbato Filho, Louise Lapagesse de Camargo Pinto, Pricila Bernardi, Angelica Francesca Maris

**Affiliations:** 1 Biologist, PhD Student in Cell Biology and Development, Universidade Federal de Santa Catarina, Florianópolis, SC Brazil; 2 Biomedic, PhD Student in Cell Biology and Development, Universidade Federal de Santa Catarina, Florianópolis, SC Brazil; 3 Biologist, PhD in Biotechnology and Molecular Biology, Laboratory Neurogene, Florianópolis, SC Brazil; 4 Biologist and MSc in Chemical Engineering, Laboratory Neurogene, Florianópolis, SC Brazil; 5 Medical Neuropediatrist, Children’s Hospital Joana de Gusmão, Florianópolis, SC Brazil; 6 Medical Geneticist and Pediatrician, Laboratory Neurogene, Florianópolis, SC Brazil; 7 Medical Geneticist, PhD in Child and Adolescent Health, Children’s Hospital Joana de Gusmão, Florianópolis, SC Brazil; 8 Medical Geneticist, University Hospital Professor Polydoro Ernani de São Thiago, Florianópolis, SC Brazil; 9 Biologist, PhD in Molecular Biology and Genetics, University Professor in the Department of Cell Biology, Embryology and Genetics, Universidade Federal de Santa Catarina, Florianópolis, SC Brazil

**Keywords:** Microarrays, Intellectual disability, Autism, Developmental disorders, SNPs, LCSH, ROH, Homozygosity

## Abstract

**Background:**

Currently, chromosomal microarrays (CMA) are recommended as first-tier test in the investigation of developmental disorders to examine copy number variations. The modern platforms also include probes for single nucleotide polymorphisms (SNPs) that detect homozygous regions in the genome, such as long contiguous stretches of homozygosity (LCSH) also named runs of homozygosity (ROH). LCHS are chromosomal segments resulting from complete or segmental chromosomal homozygosity, which may be indicative of uniparental disomy (UPD), consanguinity, as well as replicative DNA repair events, however also are common findings in normal populations. Knowing common LCSH of a population, which probably represent ancestral haplotypes of low-recombination regions in the genome, facilitates the interpretation of LCSH found in patients, allowing to prioritize those with possible clinical significance. However, population records of ancestral haplotype derived LCSH by SNP arrays are still scarce, particularly for countries such as Brazil where even for the clinic, microarrays that include SNPs are difficult to request due to their high cost.

**Methods:**

In this study, we evaluate the frequencies and implications of LCSH detected by Affymetrix CytoScan® HD or 750 K platforms in 430 patients with neurodevelopmental disorders in southern Brazil. LCSH were analyzed in the context of pathogenic significance and also explored to identify ancestral haplotype derived LCSH. The criteria for considering a region as LCSH was homozygosis ≥3 Mbp on an autosome.

**Results:**

In 95% of the patients, at least one LCSH was detected, a total of 1478 LCSH in 407 patients. In 2.6%, the findings were suggestive of UPD. For about 8.5% LCSH suggest offspring from first to fifth grade, more likely to have a clinical impact. Considering recurrent LCSH found at a frequency of 5% or more, we outline 11 regions as potentially representing ancestral haplotypes in our population. The region most involved with homozygosity was 16p11.2p11.1 (49%), followed by 1q21.2q21.3 (21%), 11p11.2p11.12 (19%), 3p21.31p21.2 (16%), 15q15 1q33p32.3 (12%), 2q11.1q12.1 (9%), 1p33p32.3 (6%), 20q11.21q11.23 (6%), 10q22.1q23.31 (5%), 6p22.2p22 (5%), and 7q11.22q11.23 (5%).

**Conclusions:**

In this work, we show the importance and usefulness of interpreting LCSH in the results of CMA wich incorporate SNPs.

## Background

Long contiguous stretches of homozygosity (LCSH) can be detected by microarray platforms through probes specific for single nucleotide polymorphisms (SNPs) [1–7]. With the consolidation of the chromosomal microarrays (CMA) as a first-line investigative test for developmental disorders, as well as the development and incorporation of robust SNP matrices, several studies have related LCSH to possible cause of disorders [8–10]. With the primary goal of detecting copy-number variations, the modern SNP containing CMA platforms now enable an even more in-depth look at the individual genomic variation, which may provide evidence for diagnostic interpretation as well as insights for more accurate genetic research [6]. Detecting and analyzing genetic variations has been a useful way to characterize the genomic patterns of individual genomes and to distinguish them from the pathogenic patterns of chromosomal abnormalities shared by a group of individuals. LCSH are one of the most common types of genomic characteristics in humans, often also referred to as runs of homozygosity (ROH) [5, 10–12].

A LCSH refers to copy-number neutral (disomic) long homozygous stretch and is used here instead of LOH (loss of heterozygosity), which more accurately describes an event where heterozygosity (once present) is now absent [13] as frequently occurs in tumors of cancer patients with disseminated metastases. Minimal thresholds for LCSH are most usually about 0.5–1.0 Mbp for population studies and more conservatively 3–10 Mbp in clinical analysis [6]. LCSH are observed throughout the human genome as consequence of endogamy or evolutionary forces [7]. Although they are typical for inbred populations, LCSH are also common and unexpectedly long in the genome of outbred populations [13], with continental distribution patterns, probably characterizing regions of low recombination in the genome [14]. The recombination-rate in the human genome is an average of roughly 2,5 recombinations per chromosomal pair, with the female meiosis showing more recombinations, 41.1 (95% CI: 39.9–42.4) than the paternal meiosis with 26.4 (95% CI, 25.7–27.2), and the telomere regions representing higher recombination rates [15].

The presence of multiple LCSH ≥5 Mbp, distributed throughout several chromosomes suggests consanguinity between the individual’s biological parents [6]. However, LCSH can also arise in consequence of gross DNA gain or loss events [10]. When LCSH regions longer than ≥10 Mbp (alone or as a sum of several LCSH) are concentrated on one single autosome, the possibility of uniparental disomy (UPD) must be considered [14]. UPD is a well-known mechanism that in some cases leads to human disease, such as when it involves a chromosomal region containing genes subjected to genomic imprinting (differential methylation in maternal/paternal germline) or when a mutation causing recessive disease resides in the now homozygous uniparental stretch [4, 5, 16]. The mechanisms that result in whole chromosome UPD are consequence of the correction of a meiotic or early mitotic error. A meiotic miss-segregation that results in the loss or gain of a whole chromosome, monosomy or trisomy, with a few exceptions renders the human zygote unviable. However, evidence shows that in some cases the chromosomal number is normalized by duplicating the only existing chromosome in a so-called monosomy rescue or, in the case of an extra chromosome, a trisomy rescue, when the extra chromosome is lost. In the case of a monosomy rescue both chromosomes of the pair are from the same progenitor (UPD) and completely identical, presenting a LCSH that spans the whole chromosome (complete uniparental isodisomy). In a trisomy rescue, there is a theoretical probability of one-third that the two chromosomes that remain are from the same progenitor. However, in a trisomy rescue the UPD can be completely heterodisomic (containing the two homologous chromosomal complements of one progenitor), partially isodisomic/heterodisomic (with one or more LCSH) or completely isodisomic (a LCSH spanning the whole chromosome), depending on the moment of miss-segregation and occurrence or absence of recombination. The trisomy rescue in the early mitotic divisions of the embryo can generate a mosaic individual, with trisomic and disomic cell lines (eventually restricted to the placenta), or a marker chromosome [6].

Except in the case of a whole chromosome isodisomy, it is difficult to know whether a LCSH representing a potential UPD is a complete or a segmental chromosomal UPD. In segmental UPDs only a part of the chromosome has the same parental origin and therefore the same-gender imprinting marks. The segmental UPD has an early and often quite complex post meiotic origin and is consequence of a translocation, DNA break or others, involving a replicative DNA repair mechanism and/or rescue of a partial trisomy [17–19].

When analyzing the LCSH in a clinical cohort, the most plausible associations can be made when very long LCSH that suggest UPDs are found on a chromosome that spans genes related to classic imprinting syndromes such as Angelman’s syndrome (UPD (15) pat), Prader-Willi syndrome (UPD (15) mat), Beckwith-Wiedemann syndrome (UPD (11) pat), Silver-Russell syndrome (*UPD* (7) mat), and the less known Temple (UPD (14) mat) and Kagami-Ogata (UPD (14) pat) syndromes, all of them neurodevelopmental diseases associated with ID, autistic behavior, developmental delay and seizures [10]. It is advisable to confirm the suspected imprinting syndrome with a methylation test when the phenotype is not unambiguous, which is often the case when CMA is requested instead of a specific test for the syndrome. Additionally, any UPD might uncover an autosomal recessive (AR) mutation in the uniparental homozygous stretch, for which in the sole transmitting parent was heterozygous [4, 20, 21].

When there are many LCSH throughout the genome indicating consanguinity, the chance of inheritance of recessive monogenic disorders increases with the degree of relatedness [22], the excess of LCSH that encompass recessive disease genes modulating the degree of cognitive impairment [9]. Also, the occurrence of multiple congenital anomalies and other significant clinical problems is higher among children of first cousins (4.4%) and second cousins (3.6%), compared to unrelated parents [2, 23, 24].

The clinical interpretation is challenging for diagnostic laboratories. Knowing common LCSH in the population, which probably do not have clinical significance, can facilitate the analysis work, since it makes it possible to guide research to rare events for candidate genes [22]. There is no work about the common LCSH which segregate in the south Brazilian population, because of the high cost of SNP arrays or CMA platforms that incorporate SNP detection, therefore population-based SNP array studies are unlikely to be performed in Brazil anytime soon. In clinical setting others have registered LCSH that were considered, with some caution, as common variation, not clinically relevant to the mainly neurodevelopmental diseases being investigated, because of their high frequency (up from about 3–5%) in the samples, some of which included non-affected parents [13, 25–28]. However, it is probable that those variations are population-specific and will vary accordingly. In view of this, we decided to, in addition to analyzing the LCSH for clinical interpretation, verify if it would also be possible to identify common LCSH due to ancestral population haplotypes using the CMA results in a cohort of individuals affected with neurodevelopmental disorders (ND).

## Methods

### Ethical aspects

The project was submitted and approved by the Research Ethics Committee of the Hospital Infantil Joana de Gusmão, the children hospital of Florianópolis-SC, Brazil, under the Nr 2,339,104, and respects the guidelines and criteria established by the resolution 466/12 of the Brazilian National Health Council. Patients or their caregivers signed the Informed Consent Form to participate in the study. In cases in which it was not possible to contact the patient for any justifiable reason (loss of contact information, mainly) the data was used and a Justification of Absence of Consent was signed by the research team, ensuring the commitment to maintain confidentiality and privacy of the individuals whose data and/or information was collected in the records.

### Sample

The sample refers to the reading files of CMA and available clinical data from 430 individuals mostly children, from the south of Brazil, with (but not restricted to) developmental delay (67%), intellectual disability (41%), autism spectrum disorder (32%) and others, alone or in combination. Referred here as neurodevelopmental disorders (ND), they often were syndromic presenting associated problems such as malformations, dysmorphologies (52% had facial dysmorphias) and seizures. The gender of the individuals was 60% male and 40% female, with a mean age of 9.5 years (0 to 49 years, sd = 9.73, Mo = 4) and predominately Caucasian.

The CMA were requested by medical geneticists and neurologists for diagnostic purpose, mainly from the Joana de Gusmão Children’s Hospital, but also from the University Hospital Professor Polydoro Hernani Santhiago and from private clinics in Florianópolis (State of Santa Catarina), throughout the years 2013 to 2016 and performed by the Laboratório Neurogene (Florianópolis, Santa Catarina, Brazil).

### Selection and analysis of LCSH

**Fig. 1:**
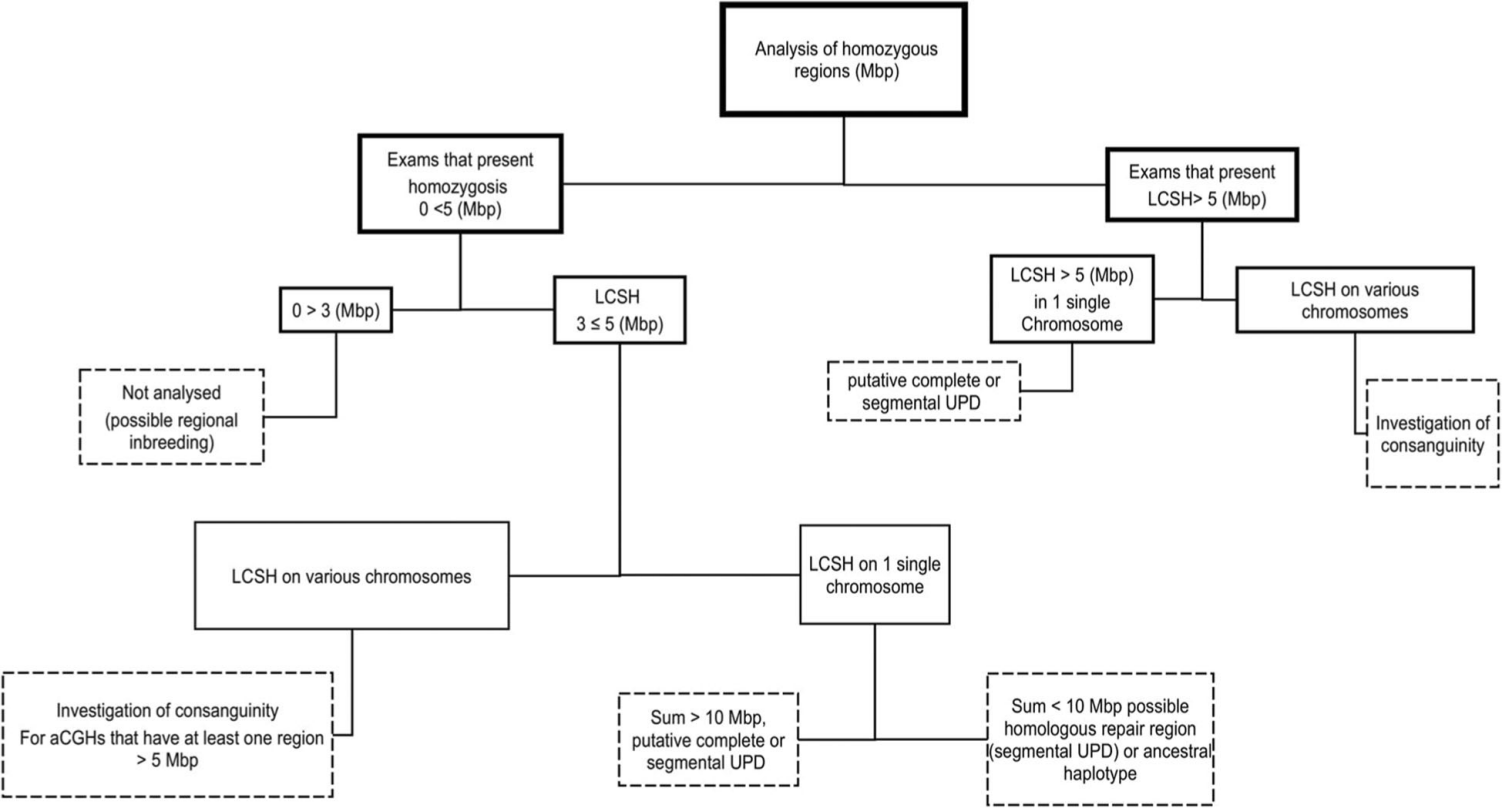
Flowchart of the analyses. Legend Fig. 1 - Flowchart depicting the steps of analyses of the exams that presented LCSHs, based on their size and distribution in the genomes of the individual

For 320 (74%) the CytoScan® HD platform was used and for 110 (26%) it was CytoScan® 750 K. The resulting files were analyzed with Chromosome Analysis Suite (ChAS) Affymetrix® software, based on the reference genome sequence of the University of California, Santa Cruz database (https://genome.ucsc.edu/cgi-bin/hgGateway), using the human genome version of February 2009 (GRCh37/hg19). The threshold for analysis of LCSH was ≥3 Mbp to unclutter the analysis, because the interest of this investigation is mainly related to clinically relevant data, where the minimal cut-off criteria usually lies between 3 and 10 Mbp and not for a populational study per se, where the cut-off threshold is considerably lower [6]. The flow of the analyses of the samples containing LCSH is illustrated in Fig. 1. All individuals with LCSH satisfying the above criteria were included, regardless of an associated presence of a pathogenic copy number variant (CNV).

### Uniparental disomy (UPD)

When the presence of LCSH was (mainly) restricted to one single autosome with a size or sum (in the case of multiple LCSH) ≥ 10 Mbp, this region was considered a possible isodisomy due to a uniparental disomy (UPD) event. A complete chromosomal UPD must be suspected even if the isodisomy is only partial, because of homologous chromosomal recombination of the internal sister-chromatids at the beginning of the meiosis (the outer sister chromatids do not recombine). This is important, then in these case genes subject to genomic imprinting do show uniparental imprinting also in the uniparental heterodisomic region of the chromosome, not only within the LCSH. This is different from segmental UPD, where only the genes within the LCSH have a uniparental imprinting pattern. Furthermore, it is also important to bear in mind that some UPDs will not have any isodisomic region, as described earlier.

### Analysis of consanguinity

The frequency of consanguinity in the cohort was calculated according to Kearney, Kearney and Conlin (2011). In short, when the homozygous pattern suggested inbreeding, all the regions of homozygosity ≥3 Mbp distributed throughout the autossomes were added; the total sum in Mbp being divided by the size of the autosome genome, 2.881 Mbp (GRCh37/hg19). The percentage obtained was correlated with the inbreeding coefficient (F) to estimate the grade of parental relationship, which is: 25%, first grade (1/4 - parent/child or full siblings); 12.5%, second grade (1/8 – half siblings, uncle/niece or aunt/nephew, double first cousins, grandparent/grandchild); 6%, third grade (1/16 - first cousins); 3%, fourth grade (1/32 - first cousins once removed); 1.5%, fifth grade (1/64 – second cousins); < 0.5% seventh grade (1/128 - third cousins), an estimation according to Kearney, Kearney e Conlin (2011). Depending on the degree of inbreeding in the population, these correlations may overestimate the direct kinship relation of the proband.

### Analysis of the most frequent LCSH

We selected and analyzed the 430 microarrays for the cytobands that most frequently showed regions with LCSH ≥3 Mbp on an autosome and those present in more than 5% of individuals were considered common LCSH. This percentage was chosen because the frequency of ≥1%, which is the usual threshold to define common polymorphisms of SNPs in a population, was not considered applicable here because this is an affected cohort. Also others have chosen the same threshold (or lower) to consider LCSH found in a affected cohort as common variation, essentially without clinical significance for their analysis [13, 25–28]. Hence, in doing so we believe to have an adequate safety margin for selecting common LCSH due to ancestral haplotypes rather than due to consanguinity or other pathogenesis-related mechanisms.

To delineate a more accurate genomic position for the most frequent LCSH, the shared homozygous sections were superimposed, and their genomic positions obtained based on the median of their beginning and end.

## Results

### LCSH in the samples

In total, 430 CMA results were analyzed whose files were available and accessible for the study. Most (95%) of the individuals presented at least one autosome LCSH (≥ 3 Mbp), resulting in a total of 1478 LCSH identified in 407 individuals. Only 23 individuals did not present a LCSH (≥ 3 Mbp). 66% of all individuals (283/430) only had LCSH under 5 Mbp (87 presenting only one and 196 with two or more), whereas 29% (124/430) presented one or several LCSH ≥5 Mbp.

### LCSH leading to suspected UPD

**Fig. 2:**
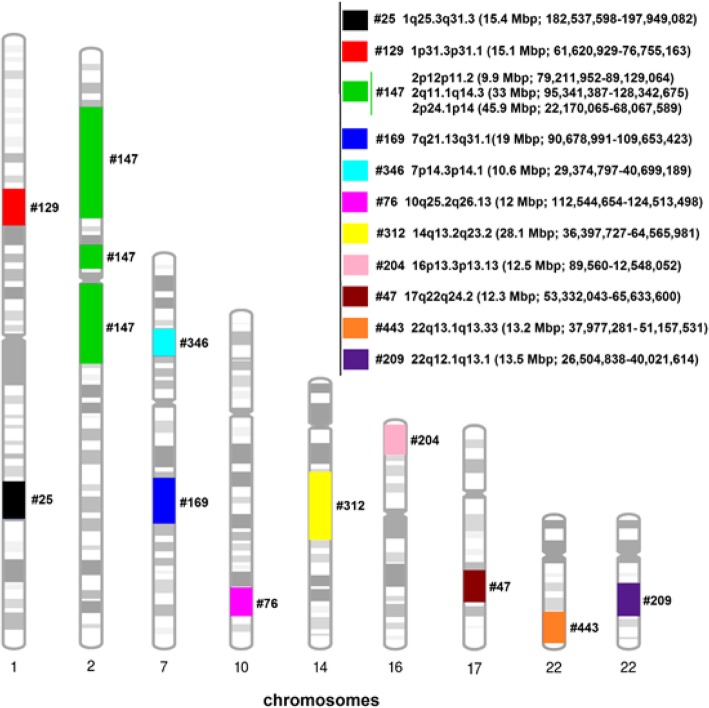
Chromosomal distribution of the 11 cases with LCSH (single or sum) ≥10 Mbp restricted to one chromosome, suggesting putative UPDs

In eleven cases (~ 2.6%) the LCSH suggested a possible UPD (Table 1 and Fig. 2).

**Table 1:** Cases with LCSH (single or sum) ≥10 Mbp restricted to one autosome suggesting a possible UPD

Case	Chromosome	UPD segment (Isodisomy)	Total Size
#25	1	1q25.3q31.3 (15.4 Mbp; 182,537,598-197,949,082)	15.4 Mbp
#129	1	1p31.3p31.1 (15.1 Mbp; 61,620,929-76,755,163)	15.1 Mbp
#147	2	2p12p11.2 (9.9 Mbp; 79,211,952-89,129,064) & 2q11.1q14.3 (33 Mbp; 95,341,387-128,342,675) & 2p24.1p14 (45.9 Mbp; 22,170,065-68,067,589)	88.8 Mbp
#169	7	7q21.13q31.1 (19 Mbp; 90,678,991-109,653,423)	19 Mbp
#346	7	7p14.3p14.1 (10.6 Mbp; 29,374,797-40,699,189)	10.6 Mbp
#76	10	10q25.2q26.13 (12 Mbp; 112,544,654-124,513,498)	12 Mbp
#312	14	14q13.2q23.2 (28.1 Mbp; 36,397,727-64,565,981)	28.1 Mbp
#204	16	16p13.3p13.13 (12.5 Mbp; 89,560-12,548,052)	12.5 Mbp
#47	17	17q22q24.2 (12.3 Mbp; 53,332,043-65,633,600)	12.3 Mbp
#209	22	22q12.1q13.1 (13.5 Mbp; 26,504,838-40,021,614)	13.5 Mbp
#443	22	22q13.1q13.33 (13.2 Mbp; 37,977,281-51,157,531)	13.2 Mbp

### Consanguinity

The analysis of the LCSH distributed throughout several chromosomes indicated some degree of inbreeding in 26.5% of the cases, with over 18% suggesting kinship of seventh to sixth grade between the parents (such as third cousins); 4.2%, fifth grade (e.g., second cousins); 1.2%, fourth grade (first cousins once removed); 2%, third grade (first cousin; half uncle with niece); 0.9%, second grade (half-siblings, uncle-niece, double cousins) and in one case (0.2%) the kinship of the parents suggested incest, since it is a coefficient of inbreeding of first grade [father (mother)/ daughter (son), full siblings]. Kinship of first to fifth grade, clinically more relevant, was suggested by ~ 8.5% of the cases. Table 2 details the results referring to the 4.3% of the cases that suggested kinship of first to fourth grade.

**Table 2:** Result of the inbreeding analysis in cases where the total sum of the LCSH ≥ 3 suggest kinship of first to fourth grade of the proband's parents

Case	∑ *of* LCSH (Mbp)	Number of chromosomes involved	Number of LCSH blocks	Possible parental relationship	Degree of kinship	Coefficient of inbreeding (F)	LCSH (IBD) expected not tested (∼%)
#194	760	19	40	father (mother) / daughter (son); complete siblings	First	0.264	25
#271	334	12	22	half-brothers; uncle (aunt)/niece (nephew); double first cousins; grandfather/granddaughter	Second	0.116	12,5
#297	314	13	25			0.109	12,5
#380	346	10	19			0.121	12,5
#220	402	18	30			0.139	12,5
#187	196	13	22	first cousins; half uncle/niece	Third	0.068	6
#275	225	10	14			0.078	6
#395	136	12	17			0.047	6
#412	123	8	12			0.043	6
#413	162	13	18			0.056	6
#419	181	9	12			0.063	6
#354	193	10	14			0.067	6
#364	165	11	15			0.057	6
#157	62	9	9	first cousins once removed	fourth	0.022	3
#273	110	13	18			0.038	3
#287	96	6	9			0.033	3
#311	82	5	8			0.028	3
#378	93	13	8			0.032	3

*IBD* Identity by descent

### LCSH with frequency ≥ 5%

Due to the scarcity of data on common LCSH in the Brazilian population, data from this population of patients with ND was explored to identify frequent LCSH in the population of Santa Catarina, potentially irrelevant to the causality of the patient’s clinical condition. The frequency of 5% or higher for considering a recurrent LCSH as common was decided in an empirical way, to allow a margin of safety in relation to the frequency of above 1% used to consider a SNP as a common variant in the population, since an affected population does present a bias. Even with this caution it cannot be ruled out that some haplotype in autozygosity acts along with other genetic variation in the manifestation of the phenotype.

**Fig. 3:**
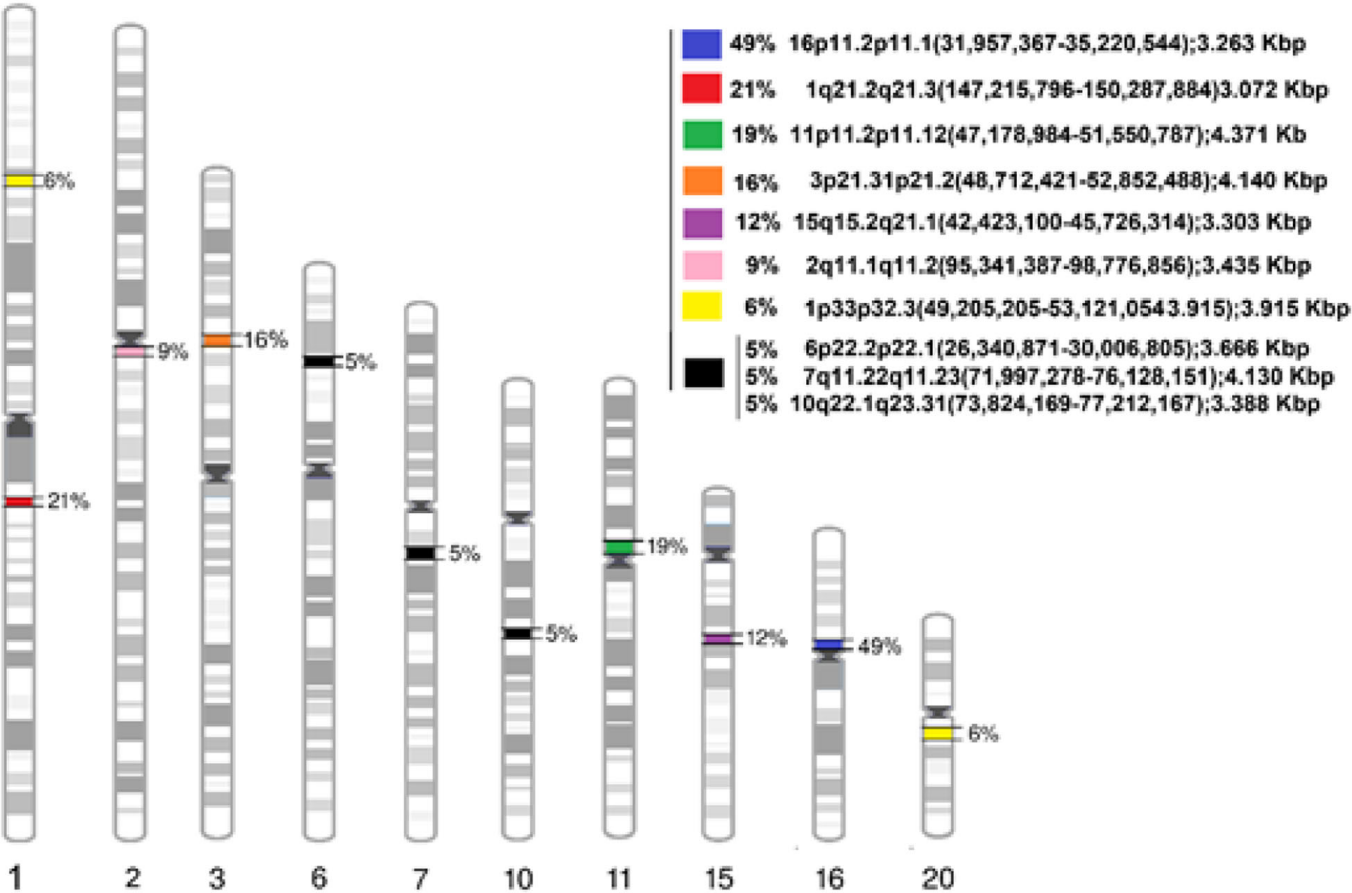
Regions of LCSH considered common (frequency ≥ 5%) identified among 430 CMA results of a cohort with developmental disorders

LCSH identified as frequent, potentially representing regions of low recombination which can maintain ancestral haplotypes that are identical by descent, are presented in Table 3 and Fig. 3.

**Table 3:** Regions of LCSH considered common (frequency ≥ 5%) identified among 430 CMA results

Frequencies	Chr/Cytobands	Initial Position	Final Position	Size (Kbp)
49%	16p11.2p11.1^a,d,e^	31,957,367	35,220,544	3.263
21%	1q21.2q21.3^d, e^	147,215,796	150,287,884	3.072
19%	11p11.2p11.12^a, d, e, b^	47,178,984	51,550,787	4.371
16%	3p21.31p21.2^a, d, e, b^	48,712,421	52,852,488	4.140
12%	15q15.2q21.1^d^	42,423,100	45,726,314	3.303
9%	2q11.1q11.2^d, e^	95,341,387	98,776,856	3.435
6%	1p33p32.3^d, e^	49,205,205	53,121,054	3.915
6%	20q11.21q11.23^d, c^	31,940,638	36,081,725	4.141
5%	10q22.1q23.31^d^	73,824,169	77,212,167	3.388
5%	6p22.2p22.1^d, e, b^	26,340,871	30,006,805	3.666
5%	7q11.22q11.23	71,997,278	76,128,151	4.130

When the beginning and/or end of the cytobands were variable, a linear position was obtained based on the median of the beginning or end. All analyses, as well as linear positions, were based on the human reference genome, version GRCh37/hg19

^a^Wang et al. 2015

^b^Pajusalu et al. 2015

^c^Ling-Hui et al. 2006

^d^Kearney H. M. (Personal communication, 2017)

^e^Sanchez P. (Personal communication, 2017)

The underlined LCSH was only found in our study

## Discussion

In the present study, LCSH suggested a frequency up to 2.6% of UPDs. The UPD frequency found by SNP microarrays varies among studies. Analyzing the results of 227 individuals neurodevelopmental disorders in a highly consanguineous population of the United Arab Emirates, Alabdullatif et al. [14], using the ISCA 4X180K platform (60,000 SNPs) suggested UPD for 1%. Sasaki et al. [29], analyzing the data of 170 parent-child trios (510 samples) for 5 HapMap populations (159 Americans from Utah, USA, with ancestry from northern and western Europe; 33 Africans with ancestry in the southwestern USA; 81 Maasai from Kinyawa, Kenya; 174 Yoruba from Ibadan, Nigeria; and 63 Mexicans with ancestry in Los Angeles, California) with the Affymetrix 6.0 Array, found regions suggesting homozygous UPD in < 1% of them. However Bruno et al. [30], found potential UPDs in 4% of the microarray samples (250 K, Affymetrix) from 117 patients of Australia with neurodevelopmental disorders, considering LCSH > 5 Mbp.

It should be noted that CMA technology only shows UPD regions in isodisomy, not detecting UPDs with total heterodisomy. Single long stretches of homozygosis, may also reflect homologous repair through a break induced DNA replication mechanism [31].

Of the eleven potential UPDs (Table 1 and Fig. 2) found in our study only three were on chromosomes related to imprinting syndromes, cases # 169 and # 346 on chromosome 7 and # 312 on chromosome 14, with two of them also carrying a pathogenic CNV, as discussed below:

Case # 169 presented an LCSH of 19 Mbp in the chromosomal region 7q21.13q31.1 (90,678,991-109,653,423). It refers to a girl, 9 years old at the time of the exam, with learning difficulties, non-specific dysmorphisms, ophthalmopathies, and short stature. However, she also had a 15 Mbp deletion involving the entire small arm of chromosome 18 [18p11.32p11.21 (136,226-15,181,666) × 1], which results in monoallelic loss of more than 184 genes, including 55 genes listed in the OMIM database, undoubtedly a pathogenic CNV [32]. There was no report of intrauterine growth retardation for the patient, the most typical characteristic of UPD(7)mat, which causes Silver Russel Syndrome, therefore, if the LCSH found represents a UPD(7), it is probable that it is a UPD(7)pat, known to not cause a pathology except when it generates autozygosis for a recessive mutation [33].

Case # 346 presents an LCSH of 10.6 Mbp in 7p14.3p14.1 (29,374,797-40,699,189). The participant was a 15 years old male adolescent at the time of examination, with development delay (DD), severe intellectual disability (ID), epilepsy, short stature, absence of speech, gastroesophageal reflux and cerebellar atrophy. In the analysis of CNVs no variation of clinical relevance was detected, only the LCSH in chromosome 7 suggesting a UPD. The UPD(7)mat causing Silver Russel syndrome, besides intrauterine and postnatal growth retardation, is characterized by short stature, triangular face, as well as mild to moderate intellectual deficiency, and speech and language difficulties in a portion of the patients [33]. The speech difficulties of the participant could be consequence of the decreased expression of the FOXP2 gene, common in patients with UPD(7)mat, which causes speech apraxia [34]; otherwise, severe ID, epilepsy and cerebellar atrophy are not phenotypes associated with UPD(7)mat. Considering the hypothesis of a gene whose mutation in autozygosity could explain the patient’s phenotype, the genes present in the LCSH were analyzed. The LCSH region encompasses 151 genes, of which 47 are OMIM genes, of which 8 are involved in diseases with an AR inheritance pattern: Ehlers-Danlos syndrome (FKBP14), Hemolytic anemia (NT5C3A), Diaphanospondylodysostosis (BMPER), Primary ciliary dyskinesia (CILD6), Pyle disease (SFRP4), Glutaric acid III (C7ORF10), Bardet-Biedl syndrome (BBS9) and Trichothiodystrophy-4 (MPLKIP). Of these, only Trichothiodystrophy syndrome 4 was considered a possible cause of the patient’s available phenotype. Trichothiodystrophy 4 or Non-photosensitive Neurocutaneous Syndrome (OMIM #234050) is due to the homozygous mutation of the MPLKIP gene, a protein involved in cell growth and replication. It is a rare AR disorder with a wide variety of clinical features including cutaneous abnormalities, DD, mild to severe ID (depending on the mutation), microcephaly, short stature, ocular abnormalities and infections, which accompany the most characteristic phenotypes of trichothiodystrophy: brittle hair and low levels of sulfur [35, 36]. This result was forwarded to the patient’s physician, who shall examine whether the patient has the hallmark of this disorder, which is the brittle hair. That would give a reliable diagnostic answer without necessarily undergoing further molecular investigation.

Case # 312, suggestive of UPD(14), with an LCSH of 28.1 Mbp in 14q13.2q23.2, refers to a male adolescent, 11 years of age at the time of testing, with abnormal brain structure, speech delay, learning disability and non-specific facial dysmorphia. However, this patient also presented a microdeletion of 2882 Kbp on chromosome 22q11.21 (18,916,842-21,798,907), a pathogenic CNV that causes DiGeorge syndrome [37], which was considered to be the cause of his clinical picture. The UPD(mat)14 causes Temple syndrome, characterized by short stature, hypotonia, motor delay, precocious puberty, small hands and feet, usually a normal intellect, eventually with learning difficulties [38]. The UPD(pat)14 causes Kagami-Ogata syndrome, a serious condition characterized by marked skeletal abnormalities [39], not present in the adolescent. Given the possibility that the patient has a true UPD, it cannot be excluded without further investigation that, in addition to DiGeorge syndrome, his condition is accentuated due to an UPD(mat)14.

For chromosomes 1, 2, 10, 16, 17 and 22, where the remaining LCSH suggestive of UPD have been detected, there is no known association with imprinting syndromes related to ND in humans. Of these cases, four also presented a CNV that was considered causal, one which indicates a complex mechanism for the origin of the LCSH. For the remaining 4 cases, there is a possibility that the phenotype is caused by unmasking a AR autozygous mutation, inherited from only one of the parents. The cases are described below.

Case # 25 presented a LSCH of 15.4 Mbp in 1q25.3q31.3 (182,537,598-197,949,082), suggesting a possible UPD(1). It refers to a young male, 16 years old on the date of the examination, which presented obesity, DD, ID, speech and/or language delay and non-specific dysmorphisms. The homozygous region in question encompasses 119 genes, of which 50 are OMIM with 10 of them involved in AR disorders. However, the CMA detected a pathogenic duplication in Xq27.3q28 (arr [hg19] 146,418,810-151,604,987) × 2, including the gene FMR1 [40], that was considered the cause of the patient’s condition.

Case # 129 presented a LCHS of 15.1 Mbp at 1p31.3p31.1 (61,620,929-76,755,163) suggesting UPD(1). The participant was a 4 years old boy at the time of the test, with DD, speech delay and autism. There are 155 genes in this homozygous region, among them 54 OMIM, with 10 related to AR disorders. Congenital glycosylation disorder, type Ic (OMIM # 603147), caused by the homozygous mutation in the ALG6 gene, leads to psychomotor retardation with delayed walking and speech, hypotonia, seizures, mild to severe ID and sometimes enteropathy [41, 42]. This result was passed on to the patient’s physician, who shall examine whether the condition could explain the patient’s symptoms and, even if considered a possible cause, whole exome sequencing (WES) is advised with focus on the LCSH, more specifically, the ALG6 gene.

Case #147 refers to a 4 years old boy at the time of the test, who presented DD and autism. In this case, three blocks of LCSH on chromosome 2 were found: 2p12p11.2 (9.9 Mbp; 79,211,952-89,129,064), 2q11.1q14.3 (33 Mbp; 95,341,387-128,342,675), 2p24.1p14 (45.9 Mbp; 22,170,065-68,067,589) totaling ~ 89 Mbp suggesting strongly a UPD(2). The LCSH regions comprise 1049 genes, of which 371 are OMIM genes and 62 related to AR disorders. The informed phenotypes of this child are not specific enough to allow the suggestion of causal genes. The possibility of the homozygous segments to harbor an AR mutation was passed on to the physician. To investigate this section, the most indicated procedure would be a WES, with an analysis directed to the LCSH regions.

Case #76 refers to a boy, 12 years old at the time of the examination, with DD, mild ID, autism and nonspecific facial dysmorphia. The CMA showed a 12Mbp LCSH at 10q25.2q26.13 (112,544,654-124,513,498), a region encompassing 130 genes, of which 56 are OMIM, including 6 genes related to AR disorders. However, the etiology of the patient’s phenotypes was attributed to a pathogenic duplication found in 7q11.23 (arr[hg19]72,556,215-74,245,599)× 3, related to Williams-Beuren duplication region syndrome (OMIM # 609757) [43].

Case # 204 refers to a girl, one-year old at the time of the test, who was referred for examination because of restricted intrauterine growth, oligohydramnios, low birth weight, low stature, hypotonia, camptodactyly, DD, speech delay, facial dysmorphia (trigonocephaly, epicanthus, downslanting palpebral fissures), and atrial septal defect. The CMA showed a 12.5 Mbp LCSH at 16p13.3p13.13 (89.560–12.548,052). The suggestive finding of a UPD on chromosome 16 is quite plausible as a consequence of a trisomy rescue, since trisomy 16 is the most common autosome trisomy reported in human miscarriages, with a 1–2% incidence in clinically recorded pregnancies, unviable as a pure trisomy [44]. A mosaic trisomy 16 is possible and has been described in association with UPD(16)mat, so much that this UPD has been considered as its bioindicator. Many of the registered cases of UPD(16)mat are consequent to findings of trisomy 16 in prenatal examinations of chorionic villi. Clinical characteristics associated with UPD of 16 are heterogeneous, and the affected individuals may present intrauterine growth retardation, malformations (often severe) and dysmorphisms [45]. Scheuvens, et al. 2017 concluded that the UPD(16) probably has no phenotype by itself and that the deleterious phenotypes found are caused by an often undetectable mosaicism of the trisomy 16 (including a possible effect of a trisomic placenta).

In case # 47, a 12.3 Mbp LCSH in the 17q22q24.2 (53,332,043-65,633,600) was detected in the CMA of a girl, 8 years old at the time of the test, with short stature and anomalies of the upper and lower limbs. The LCSH suggesting the UPD(17) contains 238 genes, including 93 OMIM genes of which 15 are involved in AR disorders. However, the micro*array* in this case also showed a deletion on the X chromosome (arr[hg19]Xp22.33[679,520-950,907]× 1) involving the pseudo-autosome SHOX gene, that in haplo-insufficiency is the cause of the Leri-Weill dyschondrosteosis syndrome [46], which was considered the cause of the patient’s # 209 refers to a boy, 5 years old at the time of the examination, presenting DD and speech delay. A 13.5 Mbp LCSH at 22q12.1q13.1 (26,504,838-40,021,614), suggestive of UPD(22) was detected on the patient’s CMA. The LCSH encompasses 289 genes, among these 154 OMIM, with 17 genes related to AR disorders, however no obvious candidate gene was identified. WES with focus on the LCSH region is advised.

Case # 443 refers to a 2-year-old boy referred for examination with low weight, low stature, DD, Mongolian spots, poor ear formation, speech delay, autism, aggression and behavior. The genomic findings of this case call attention to the peculiarity of having a region of approximately 13.2 Mbp in homozygosis on chromosome 22q13.1q13.33 (37,977,281-51,157,531), accompanied by a micro-triplication of 2.8 Mbp resulting in four copies of the affected segment 22q12.3q13.1(35,888,588-38,692,765)× 4, which includes part of the homozygous region. Additionally the CMA also reveals a mosaic gain in the homozygous regions 22q13.1q13.33 (37,933,985-51,197,766)× 2–3. Two similar cases with an interstitial triplication (resulting in four copies) followed by uniparental isodisomy (isoUPD) for remainder of the chromosomal arm were described earlier [47]. The authors explained the origin of this type of alteration through a microhomology-mediated break-induced replicational DNA repair mechanism inducing copy number gains and segmental isoUPD in tandem. However, in the presented cases the breakpoint was the same for the copy-number gain and the isoUPD, whereas in our case the tetrasomic region is partially isozygotic (for about 650 Kb). In addition, virtually the whole LCSH segment is in a 2–3 copy mosaicism, challenging the understanding of the mechanism that originated this alteration, although mosaicisms are occasionally found with genetic alterations and with UPD mosaicisms [48]. It seems likely that the much-increased gene dosage is responsible for the patient’s phenotype, however the contribution of an AR mutation in autozygosis cannot be ruled out.

The LCSH detected by CMA with SNPs are mostly explored to communicate to the requesting clinician the possibility and probable grade of parental kinship when excessive homozygosity is found, to alert about the higher possibility of a recessive disorder and the risk of recurrence for future pregnancies. They still are not widely explored in the clinical analysis to investigate potential UPD and eventual derived imprinting disorders or for the search for genes related to AR disorders. The main reason for this is that the interpretation of single or a few LCSH is very speculative and most findings will require further molecular investigations, for instance, methylation analysis in case of suggestive UPD in an imprinted chromosome. In some cases, the in silico analysis of the disease-causing recessive genes in the LCSH region will indicate a clear candidate gene whose mutation can explain the clinical features of the patient. For most cases, however, when the phenotype is not very pronounced and/or the LCSH region contains a high amount of disease-causing genes, WES would be the logical option, since the LCSH directs the focus of analysis, with a higher probability of identification of the causal gene [6, 48, 49]. However, in Brazil and most other countries, WES is a high-cost exam that rarely is covered by medical insurance.

The LCSH pattern of ~ 18% of the individuals indicated distant (sixth or seventh grade) descent, which may be due to regional characteristics of immigration and marriages within the same ethnic group of immigrants in the south of Brazil. In the State of Santa Catarina there is a substantial German and Italian immigration, and until recently there was well known resistance among Germans and Italians to marry outside their ethnic groups and religious beliefs, especially in the rural region. When the kinship indicated by the LCSH is distant and more related to endogamic characteristics of the population, it decreases the probability of a clinical relevance.

For 8% of the individuals, the LCSH suggested a parental relationship from first to fifth grade; these are more likely to suffer a clinical impact, since the closer the kinship the greater the proportion of shared alleles, and therefore the risk of inheriting two copies of an AR mutation [6]. When the parental relationship is very close, such as second or even first grade, the patient is most likely affected by an AR mutation. However, the extraordinary high percentage of homozygosity in their genomes (12 to 25%) does not greatly restrict the target region in the genome to speculate for about causal genes, and WES will be the most likely tool to identify the mutation.

In clinical setting the greatest difficulty in analyzing LCSH is not to know if they are relevant enough to be analyzed or reported. When they are so numerous as to indicate parental consanguinity, this excess of homozygosity usually is reported. When the analysis of LCSH goes further than that, those that suggest UPD are also reported. The greatest doubt is what to do with LCSH that are not as large as to raise a UPD hypothesis, but still, are over 3–5 Mb. Should they be ignored or checked for recessive genes? To do that with every sample is very time consuming and very speculative. Knowing which LCSH are common and potentially can be considered a characteristic of the population, allows to focus the analysis on the most relevant LCSH. And that seems to be the approach of groups which analyze larger samples. The LCSH that are found recurrently in patients and unaffected parents are considered common variation and ignored from a certain point on [13, 25–28]. Following the same rationale and using similar criteria, LCSH ≥3 Mbp found in a frequency of 5% or higher, we identified 11 LCSH that were considered as common variation in our population and now report them to contribute with the growing evidence.

Of the LCSH identified as frequent in our data-set (Table 3) all, except 7q11.22q11.23 (71,997,278 -76,128,151), have been found to be common LCSH by other groups in clinical investigation of patients with developmental disorders [25–28]. Those LCSH are assumed to represent regions of low recombination (ancestral haplotype blocks) and interpreted as potentially nonpathogenic.

The common LCSH regions found in our population, 16p11.2p11.1 (31,957,367-35,220,544), 11p11.2p11.12 (47,178,984 -51,550,787) and 3p21.31p21.2 (48,712,421-52,852,488) were also reported by Wang et al. (2015) [26] as recurrent LCSH with no clinical relevance in a cohort of patients with ND including unaffected parents; by Kearney H. M. [28] in a frequency > 5% in CMA (CytoScan® HD, Affymetrix) reads from affected individuals; and also by Sanchez P. [27], who considered common LCSH to be at > 3% frequency in the CMA (CytoScan® HD, Affymetrix) samples from a cohort of 278 affected Hispanics. Pajusalu et al. (2015), also reported the same regions on chromosome 3 and 11 as recurrent LCSH with a frequency of 9.3 and 6%, respectively, using as minimal cut-off the size of 5 Mb, in the investigation of 2110 consecutive Estonian patients (including pre-natal samples and parents).

The regions 1q21.2q21.3 (147,215,796-150,287,884), 2q11.1q11.2 (95,341.387-98,776,856), 1p33p32.3 (49,205,205-53,121,054) and 6p22.2p22.1 (26,340,871-30,006,805) were not reported by Wang [26], but are common findings in the Kearney H. M. [28] and Sanchez P. [27]. The region on chromosome 6 was also a common finding (4.9%) by Pajusalu et al. (2015). Moreover, our common LCSH on 20q11.21q11.23 (31,940,638-36,081,725) was also reported by Sanchez P. [27] and Pajusalu et al. (2015). Finally, regions 15q15.2q21.1 (42,423,100-45,726,314) and 10q22,1q23,31 (73,824,169-77,212,167), were only reported previously in the samples of Sanchez P. [27].

We found no previous report of the LCSH at 7q11.22q11.23 (71,997,278 -76,128,151), encountered in the population of Santa Catarina at a frequency of 5%. This homozygous region is not related to any gene with known imprinting pattern in humans [50] and covers 102 known genes, of which 46 are listed on OMIM, including with 3 genes related to AR disorders: a less common form of chronic granulomatous disease (#233700), Antley-Bixler syndrome with genital anomalies and disordered steroidogenesis (# 201750), and also disordered steroidogenesis due to cytochrome oxidoreductase P450 (# 613571).

The LCSH considered as frequent and common in the present study partially corroborate studies of Yang et al. [7], who emphasized that frequent regions of LCSH occur in the vicinity of centromeric gaps, exemplifying their findings on chromosomes 8, 9, 11, 16, 19 and 20 in the Asian population, and chromosomes 8, 9, 11, 16 and 19 in the Caucasian population. Our study found common LCSH in the vicinity of the centromeric gaps in above-mentioned chromossomes 11 and 16, but also on chromosomes 1, 2, 7 and 20, and intersticial LCSHs on 1p, 3p, 6p, 10q and 15q (Fig. 3).

As mentioned before, athough a pathogenic implication of the LCSH reported as common seems unlikely, these results should be considered with caution, in particular because we have no knowledge of the haplotypes that are segregating. To affirm unambigously that the recurrent LCSH found in affected cohorts have no clinical implication, they should appear in similar frequencies in the non-affected population. This only will be known in the future, as data from affected and normal populations accumulate. Haplotype investigation of common LCSH found in our cohort is a future goal and will provide additional relevant information.

## Conclusions

In this cohort of 430 individuals with ND, 11 LCSH > 10 Mbp ranging from 10.6–88.8 Mbp (isolated or as a sum) were found restricted to a single autosome, indicating a potential frequency of UPDs of 2.6%. However, the limitation imposed by the CMA only enables us to suggest UPD when uniparental isodisomy is present, which may underestimate the UPD rate. Since no methylation tests were performed, it cannot be stated that all these findings represent actual UPDs nor differentiate complete from segmental UPD. Of the 11 cases that presented a potential UPD, 6 presented a pathogenic quantitative alteration (CNV) that explained at least most of their clinical condition, which included two of the three cases that could potentially represent imprinting disorders. The identification of the extensive homozygous region in a single chromosome, even when it does not suggest imprinting syndrome and there is no other alteration that may explain its condition, represents a target region with potential to harbor a causative AR autozygous mutation, and these patients can be forwarded to WES.

Consanguinity suggesting descent from first to fifth grade was found in about 8.5% of the individuals. In these cases, it is important to advise the parents about the empirical risk of recurrence, considering the possibility that the cause is a AR autozygous mutation, with greater risk when the degree of kinship is high. If there are more affected siblings check on the possibility of performing an analysis of the identical by descent regions to narrow down the region with the causative gene, also for eventual WES.

Finally, a series of 11 LCSH are reported that are present in a frequency above 5% in individuals with affected neurodevelopment, 10 of them reported as common by other groups, suggesting that those really are potential normal variations in the population of Santa Catarina. This information is valuable in facilitating the selection of LCSH most likely to be of clinical relevance for analysis, in particular in the context of single LCSH shorter than 10 Mbp which do not are candidates for UPD. The possibility of these LCSH harbor a disease-causing mutation cannot be completely ruled out.

The investigation of LCSH using the information obtained by CMA with high SNP density represents helpful information to aid the investigation of neurodevelopmental disorders. Nevertheless, without the possibility of performing complementary molecular tests such as methylation analysis, the sequencing of a specific gene or WES, these findings are mostly theoretical and suggestive. The LCSH analysis provides an investigation-guide, but usually cannot deliver by itself a definitive diagnostic outcome.

## Abbreviations

AR: Autosomal recessive
CMA: Chromosomal microarrays
CNV: Copy number variant
DD: Development delay
F: Inbreeding coefficient
ID: Intellectual disability
LCSH: Long contiguous stretches of homozygosity or runs of homozygosity
ND: Neurodevelopmental disorders
OMIM: Online Mendelian Inheritance in Man databank
SNP: Single nucleotide polymorphisms
UPD: Uniparental disomy
WES: Whole exome sequencing
